# Is There a Role for Dual PI3K/mTOR Inhibitors for Patients Affected with Lymphoma?

**DOI:** 10.3390/ijms21031060

**Published:** 2020-02-05

**Authors:** Chiara Tarantelli, Antonio Lupia, Anastasios Stathis, Francesco Bertoni

**Affiliations:** 1 Institute of Oncology Research, Faculty of Biomedical Sciences, USI, 6500 Bellinzona, Switzerland; chiara.tarantelli@ior.usi.ch; 2 Department of Health Sciences, University “Magna Græcia” of Catanzaro, 88100 Catanzaro, Italy; lupia@unicz.it; 3 Oncology Institute of Southern Switzerland, 6500 Bellinzona, Switzerland; anastasios.stathis@eoc.ch; 4 Faculty of Biomedical Sciences, USI, 6900 Lugano, Switzerland

**Keywords:** TORC1, TORC2, PI3K, lymphoma, chronic lymphocytic leukemia

## Abstract

The activation of the PI3K/AKT/mTOR pathway is a main driver of cell growth, proliferation, survival, and chemoresistance of cancer cells, and, for this reason, represents an attractive target for developing targeted anti-cancer drugs. There are plenty of preclinical data sustaining the anti-tumor activity of dual PI3K/mTOR inhibitors as single agents and in combination in lymphomas. Clinical responses, including complete remissions (especially in follicular lymphoma patients), are also observed in the very few clinical studies performed in patients that are affected by relapsed/refractory lymphomas or chronic lymphocytic leukemia. In this review, we summarize the literature on dual PI3K/mTOR inhibitors focusing on the lymphoma setting, presenting both the three compounds still in clinical development and those with a clinical program stopped or put on hold.

## 1. Introduction

The activation of the phosphoinositide 3-kinase (PI3K)/v-akt murine lymphoma viral oncogene homolog (AKT)/mammalian target of rapamycin (mTOR) pathway is a main driver of cell growth, proliferation, survival, and chemoresistance of cancer cells [1,2,3,4,5]. The PI3K/AKT/mTOR pathway has already represented an attractive target for developing targeted anti-cancer drugs for many years, and, indeed, many isoform-specific, pan-inhibitors, or dual PI3K/mTOR inhibitors have already entered the clinical evaluation and also undergone approval by regulatory agencies [1,2,3,4]. The pathway is also important for lymphoma cells and its pharmacological inhibition has shown clinical benefit for patients that are affected by different lymphoproliferative neoplasms [1,2,6]. 

The present review will focus on dual PI3K/mTOR inhibitors in clinical and pre-clinical development for patients that are affected by hematological malignancies after providing an overview of the pathway in relation with cancer.

## 2. The PI3K Signaling Pathway

Human cells express three classes of PI3Ks proteins, according to primary sequence homology, regulation, and *in vitro* substrate specificity [1,3,4,7,8]. PI3Ks are composed by a catalytic isoform complexed with a regulatory subunit, which regulates the activity, localization, and binding of the dimer. In mammals, class I PI3Ks are divided into IA and IB subclasses that are based on their regulation criteria. Class IA PI3Ks includes heterodimers of p110 catalytic subunit and p85 regulatory subunit. The class IA catalytic subunit isoforms are encoded by the genes *PIK3CA*, *PIK3CB*, and *PIK3CD* (p110α, p110β, and p110δ, respectively). These isoforms can associate with any of five regulatory isoforms, p85α and its splicing variants p55α and p50α (*PIK3R1*), p85β (*PIK3R2*), and p55γ (*PIK3R3*), generally called p85 type regulatory subunits [7]. Class IB PI3Ks are heterodimers of a p110γ catalytic subunit (*PIK3CG*) with regulatory isoforms p101 (*PIK3R5*) or p87 (*PIK3R6*). The catalytic isoforms p110α and p110β are ubiquitously expressed, while p110δ and p110γ are mainly expressed in leukocytes, but also in other tissues, such as heart, pancreas, liver, and skeletal muscle [8]. Class IA are activated by receptor tyrosine kinases (RTKs), while G protein-coupled receptors (GPCR) activate class IB PI3Ks. The phosphotyrosines in the RTK consensus YxxM sequence can physically interact with the regulatory subunit or indirectly through adaptor proteins, such as IRS1 (insulin receptor substrate) and IRS2, leading to the autophosphorylation of tyrosines by RTK homodimerization and the recruitment of PI3K to the plasma membrane [7]. Ras (Rat sarcoma) can activate class I PI3Ks by direct binding to the p110 catalytic isoform [3]. Class II PI3Ks solely consist of the catalytic subunit, which has three isoforms PI3K-C2α, PI3K-C2β, PI3K-C2γ (encoded by *PIK3C2A*, *PIK3C2B*, *PIK3C2G*), while using phosphatidylinositol 4-phosphate (PI(4)P) as substrate [8]. Class II PI3Ks regulate angiogenesis, cellular growth and survival, but they are less characterized than class I PI3Ks [8]. Class III PI3Ks is composed by a single *PIK3C3* gene, translated in the VPS34 (vacuolar protein sorting-associated protein 34) protein, which forms a heterodimer with VPS15 (encoded by *PIK3R4*) and produces phosphatidylinositol 3-monophosphate (PI(3)P) [9]. The VPS34-VPS15 dimer is implicated in intracellular trafficking and autophagy [10].

**Figure 1:**
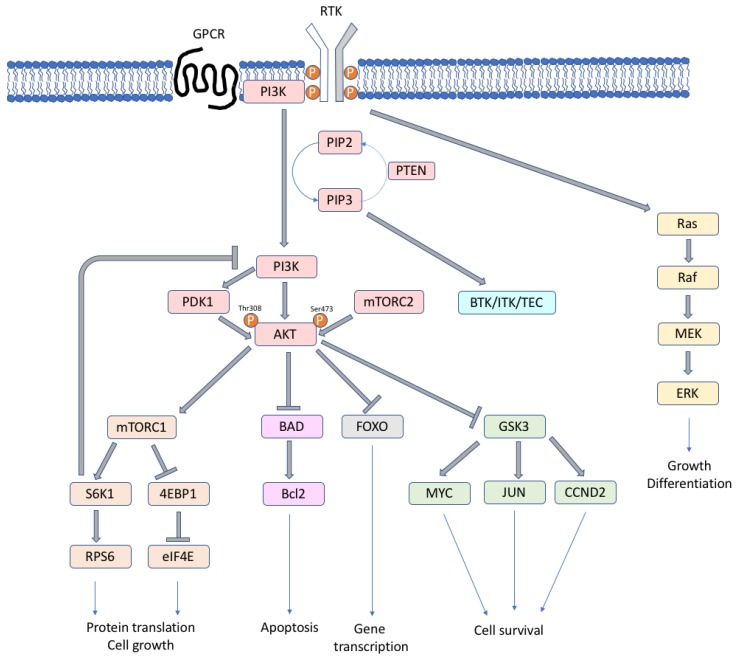
Simplified scheme of the PI3K/mTOR signaling cascade.

The various forms of PI3K play different roles, among them memory storage and retrieval [11,12], metabolism and insulin signaling [13], and immunity [14]. The activated form of the lipid kinases PI3Ks phosphorylates the 3′-hydroxyl group of plasma membrane phosphoinositides (PtdIns), producing three types of second messengers: phosphatidylinositol 3-monophosphate (PI(3)P), phosphatidylinositol 3,4-biphosphate (PI(3,4)P_2_), and phosphatidylinositol 3,4,5-triphosphate (PI(3,4,5)P_3_/PIP_3_) [7]. The PIP_3_ levels are regulated by PTEN (phosphatase and tensin homolog), which is an important tumor suppressor, with phosphatase activity that is able to convert PIP_3_ to PI(3,4)P_2_. When the second messenger PIP_3_ is formed, downstream PI3K targets, such as AKT and mTOR, are activated (Figure 1). PIP_3_ binds and phosphorylates AKT at Ser473 by the mammalian target of rapamycin complex 2 (TORC2) and at Thr308 by phosphoinositide-dependent protein kinase-1 (PDK1) [7]. The activation of AKT is maximal when both sites are phosphorylated, and it leads to the phosphorylation of large spectra of proteins that are involved in cell growth, survival and progression, protein synthesis, and metabolism [7,8,15]. AKT can activate TORC1 complex, which regulates ribosomal protein S6 kinase 1 (S6K1, or p70S6K), eukaryotic translation initiation factor 4E-binding protein 1 (4EBP1), two key regulators of protein synthesis [16], RAS/ERK, forkhead/winged helix box class O (FOXO) family, and other pathways. AKT inhibits the apoptotic pathway by negatively regulating the pro-apoptotic Bcl2 family proteins and mediating p53 degradation. In lymphocytes, the TEC family tyrosine-kinase proteins BTK (bruton tyrosine kinase), ITK (IL2 inducible T-cell kinase), and TEC are PI3K effectors that are activated by PIP_3_, with potential anti-tumor therapeutic implications. TORC1 or S6K1 are involved in a negative feedback regulation loop; their activation leads to the deactivation of the PI3K, AKT, and ERK pathway [17,18].

## 3. PI3K Pathway and Metabolism

PI3K signaling is activated upon grow factor stimulation and grow factor receptors activation, including insulin receptor (INSR), which regulates metabolic homeostasis. Conformational changes of the activated receptors allow autophosphorylation and the activation of INSR, which recruits IRS proteins, which are phosphorylated by INSR and finally create the binding motif for p85 [3,19]. The early insulin-driven PI3K signaling leads to enhanced glucose transporters (GLUT) translocation in the membrane [20] and augmented transcription and translation of genes coding for these transporters [21], increasing the glucose uptake in muscle and fat cells. The isoform p110α mediates glucose homeostasis in muscle, liver, and fat [22]. The regulatory subunit p85 could act both as a positive and negative regulator of insulin signaling. In fact, p85 could also block IRS signaling [23], which causes the inhibition of insulin signaling and formation of insulin resistance. Based on its normal biologic functions, it is obvious that pharmacological targeting the of PI3K/mTOR pathway as cancer treatment is unlikely to be devoid of on-target metabolism-related toxicities. Indeed, drugs that target p110α induce acute insulin resistance, which causes severe hyperglycemia, leading to hyperinsulinemia [3].

## 4. Deregulation of the Signaling in Cancer

As already mentioned, a constitutive activation and deregulation of the PI3K/AKT/mTOR pathway is almost a hallmark of cancers cells [1,2,4,24,25]. Somatic mutations in the genes encoding for the catalytic PI3K isoforms (i.e., *PIK3CA*) or regulatory isoforms (i.e., *PIK3R1*) are frequent [24,26,27]. Mutations targeting catalytic class I PI3K isoforms are only associated to *PIK3CA* catalytic isoform, while in the other class I catalytic isoforms *PIK3CB, PIK3CD*, and *PIK3CG* tumor associated mutations are very rare [28,29]. Mutations in the *PIK3CA* are associated with augmented kinase activity and cluster in two ‘hotspots’, one in the exon 9 (E542K and E545K, in the helical domain), the other in exon 20 (H1047R, in the kinase domain) [28,30,31]. The E542K and E545K mutations disrupt the inhibitory interface with the regulatory subunit p85, while H1047R mutation enhance the interaction of the kinase domain with cell membranes. Nevertheless, proteins p100β, p100δ, and p100γ are also capable of inducing oncogenic transformation in their wild-type form [32], in line with the fact that *PIK3CB*, *PIK3CD*, and *PIK3CG* are generally amplified or overexpressed, but not mutated, in cancer. Genes encoding AKT isoforms, mTOR, and PTEN are targeted by somatic mutations deregulating the PI3K/AKT/mTOR pathway. Mutations in the RTK or RAS genes, RTK receptor overexpression/amplification, autocrine loops involving RTKs, and the ligands are also recurrent events [6].

The regulatory isoforms p85 are also involved in tumorigenesis. Specific *PIK3R1* mutations in the region of the protein that interacts with p110 stabilize the p110 isoforms and abrogate the inhibitory action of p85 on p110 [33]. Mutations in other PI3K regulator subunits are rare. 

The amplification of *AKT1* and *AKT2* and activating mutation of *AKT1* (in E17K) increase AKT1 binding to the plasma membrane and its phosphorylation in solid tumors [34]. 

A study investigated the somatic alterations involving the PI3K/AKT/mTOR pathway in pan-cancers, finding mutations or copy number alterations in *PIK3CA* (14% mutated, 6% amplified), *PTEN* (9% mutated, 7% deletion or two-hit loss), *PIK3R1* (4% mutated), *AKT1* (1% mutated, 3% amplified), and *MTOR* (4% amplified) [24]. In 5% of cases, genomic rearrangements in *PTEN* and *PIK3R1* have been found. Mutations in *AKT1*, *MTOR*, *PIK3CA*, *PIK3R1*, and *PTEN* with predicted functional effects have higher phospho-AKT levels when compared to tumors with no alteration, and the *PTEN* copy losses were associated with AKT activation. They also found an association between *STK11* mutation, *STK11* copy loss, *PTEN* copy loss, *PIK3CA* amplification, higher phospho-4EBP1 expression, and worst patient outcome. Interestingly, mutations that were related to RTK signaling were not associated with PI3K/AKT/mTOR pathway activation.

## 5. Deregulation of the Signaling in Lymphoma

The PI3K pathway is activated by a large number of mechanisms across B-cell malignancies. In patients, *PIK3CA* mutations or amplification were found, respectively, in 8% of DLBCL (diffuse large B-cell lymphoma), mainly in the catalytic domain, [35,36], and in 68% of mantle cell lymphoma (MCL) [37,38]. Chronic lymphocytic leukemia (CLL) patients rarely have *PIK3CA* mutations [39] and amplification of *PIK3CA* has been reported in 5.6% of the cases [40]. *PTEN* loss was observed in 15% of MCL patients [37,38], in 37–55% of DLBCL patients [35,41,42], and in 21% of follicular lymphoma (FL) [43]. Low levels of *PTEN* were observed also in CLL patients [44]. Among DLBCL, the loss of *PTEN* expression was found in 55% of GCB (germinal center B-cell type) DLBCL patients, and only in 14% of non-GCB DLBCL cases [41]. In GCB DLBCL cell lines and primary patient samples, *PTEN* status was inversely correlated with the activation of the PI3K/AKT pathway, suggesting that activation of this pathway could give an oncogenic addiction for this subtype of DLBCL. In fact, in GCB DLBCL, deletions of *PTEN*, and amplification of the MIR17HG (microRNA-17-92 cluster) sustain cell proliferation [45]. Similar deregulation of PTEN and MIR17HG is also in place in Burkitt lymphoma (BL) cells, in which the constitute activation of the *MYC* oncogene that is due to chromosomal translocation also directly activates PI3K/AKT/mTOR [46,47,48,49]. 

PI3K pathway activation can also be mediated by B cell receptor (BCR) signaling. The phosphorylation of CD19 by BCR leads to the binding of the regulatory subunit p85, and the recruitment of the p110 catalytic subunit [50]. BTK, which is a downstream protein of BCR, is activated by PI3K (by PIP3) in B cells. 

Recurrent mutations in *PIK3CD*, *PIK3R1*, and *MTOR* occur in DLBCL primary tumors [51]. Three cases were characterized by single point mutation T → G in the catalytic domain of *PIK3CD*, converting Asp (uncharged side chains) in Lys (charged side chains). The mutations in *PIK3CD*, *PIK3R1*, and *MTOR* did not all cluster in a single hot spot of the gene, but they were spread across it. *PIK3CD* mutations were localized in the catalytic domain (N948K, E1021K), *PIK3R1* mutations occurred in the RhoGAP-p85 domain (V172M, A210D), and in the COG4942 domain (Y470D, D560G), *MTOR* mutations were in the HEAT domain (A835T), close to the FAT and rapamycin binding domains (N1765D, A37T, T182K), and to the FATC domain (V619I). The cell lines with *MTOR* mutations showed a higher sensitivity to PI3K inhibitor than those that did not harbor *MTOR* mutations, which suggests that sensitivity to PI3K inhibitors correlates with *MTOR* mutations. 

In GCB DLBCL cell lines, a tonic (antigen-independent) BCR signaling activates AKT, regulates proliferation and size with different magnitudes, and *AKT* knockout resulted in toxicity [52].

Chronic active BCR signaling is typical of ABC (activated B cell) DLBCL, the subgroup of DLBCL with constitutive activation of NFκB (nuclear factor κB), also regulated by PI3K [53].

The phosphorylation of AKT was detected in 52% of DLBCL [54], and the sensitivity to AKT inhibition correlated to the efficacy of the inhibitor to block phosphorylation of S6K1 and RPS6 [55]. Cell lines expressing AKT-independent S6K1, as activated by upregulation of PIM2 (Pim-2 Proto-Oncogene, Serine/Threonine Kinase) or activation of BCR, are resistant to AKT inhibitors. Combining AKT inhibitor with BTK, PIM2, or S6K1 inhibitors could overcome resistance to AKT inhibition in this subtype of lymphoma.

Finally, the PI3K/AKT/mTOR signaling also plays an important role in the different non-neoplastic cells that are present in the tumor microenvironment, for example regulating the proliferation and migration of endothelial cells, the M1/M2 polarization of macrophages, and the activation and/or differentiation of T cells [56,57,58,59,60,61,62]. 

## 6. Dual PI3K/mTOR Inhibitors in Lymphoma

We can recognize different classes of compounds targeting the PI3K/AKT/mTOR pathway: pan-PI3K inhibitors, isoform specific inhibitors (i.e., idelalisib), dual PI3K/mTOR inhibitors, AKT inhibitors, allosteric mTOR inhibitors (rapalogs) inhibitors, and mTOR kinase inhibitors [1,2,4,14,63,64,65,66,67,68,69]. An interesting strategy is to target several PI3K isoforms as well as mTOR instead of a single PI3K isoform or only mTOR. In fact, the catalytic isoform of the p110 subunit and mTOR have structural similarities, and targeting two crucial points of the same pathway could lead to higher efficacy, could overcome feedback inhibition coming from mTOR inhibition [70], and the risk of drug resistance that should easily come out in the case of treatment with compounds targeting a single p110 isoform [71,72,73]. Indeed, the dual PI3K/mTOR inhibitors have shown, at least in the preclinical setting, an improved than what achieved targeting individually single PI3K isoforms, all PI3K isoforms or mTOR [74,75,76,77]. Table 1 shows an exhaustive list of the dual class I PI3K and mTOR inhibitors. Table 2 summarizes the clinical data available for patients that are affected by lymphoma enrolled in phase I/II studies with such a class of compounds. The chemical structures, International Union of Pure and Applied Chemistry (IUPAC) names, and molecular weights for all of the compounds are shown in Table 3 or Table S1, depending on their current clinical development stage. Indeed, there are only three dual PI3K/mTOR inhibitors that are still in clinical development for humans: bimiralisib, GDC-0084, and gedatolisib. 

**GDC-0084** (RG 7666) is orally given and it passes the blood brain barrier (BBB) and it has been specifically developed for patients with brain tumors or with brain metastases from solid tumors [87,88,89]. **Gedatolisib** (PF05212384/PKI-587) is an intravenous inhibitor of PI3Ks (preferentially of p110α, also acting on the mutant forms) and TORC1/TORC2 with preclinical activity in different solid tumor models [90,91]. There are no data in lymphoma models, while the compound has been extensively tested in lymphoblastic and myeloid leukemia models [92,93,94,95]. No lymphoma patient was treated in the phase I study [96].

**Bimiralisib** (PQR309) inhibits all of the PI3Ks (p110α, first, followed by p110δ, p110β, and p110γ) and TORC1/TORC2 [97]. It is an oral compound and it is capable of crossing the BBB [97]. When comparing the published data [97,98], bimiralisib has an overlapping inhibition of p110α with voxtalisib, a reduced inhibition of p110δ, p110β, and p110γ, a higher activity on mTOR, and a reduced activity on the off-target DNA-PK [97]. Bimiralisib has *in vitro* and *in vivo* anti-lymphoma activity in models that were derived from B and T cell lymphomas [75], and also from canine DLBCL [99]. The pattern of activity is highly correlated to what was achieved by apitolisib, another dual PI3K/mTOR inhibitor (see below), but bimiralisib shows higher IC50 values [75]. Bimiralisib also shows *in vitro* activity in lymphoma cell lines with primary or secondary resistance to the PI3Kδ inhibitor idelalisib [75]. Analysis of transcriptome and phospho-proteomics of DLBCL cells that were exposed to bimiralisib demonstrates that the compound modulates transcripts and proteins that are involved in fundamental pathways and signaling cascades: BCR signaling, NFκB pathway, PI3K/AKT/mTOR pathway, mRNA processing, apoptosis, cell cycle, MAPK/RAS signaling, Myc pathway, and glycolysis [75]. Moreover, the modulation of these pathways occurs via changes at both the RNA and protein phosphorylation levels. However, while the mTOR pathway and mRNA metabolism are more regulated at the protein level, cell cycle and BCR signaling changes are more driven by expression level changes [75]. Interestingly, the early changes at the RNA level seen after bimiralisib exposure are largely overlapping with what was observed after exposing the same DLBCL cell lines to the p100δ inhibitor idelalisib, the p100δ/ p100γ inhibitor duvelisib, or the BTK inhibitor ibrutinib [75], in accordance with studies that were performed in normal B cells after BTK and PI3K genetic silencing [100]. Importantly, an adaptive mechanism with an early upregulation of genes coding members of the BCR signaling is induced by bimiralisib, idelalisib, duvelisib and ibrutinib, partially explaining the benefit of targeting the BCR signaling with compounds targeting multiple of its members [75]. Bimiralisib as well as other BCR signaling inhibitors, up-regulates CXCR4, which is a possible marker of adaptive resistance [75,101].

Following the completion of a phase 1 study in patients with advanced solid tumors [102], bimiralisib has entered the clinical setting for patients with relapsed/refractory lymphoma (phase I/II: II NCT02249429, NCT03127020), and for patients with relapsed/refractory primary central nervous system lymphomas (NCT02669511). Unfortunately, the only data publicly available refer to the first 15 patients that were enrolled in the phase I (NCT02249429). Reported severe (G3/G4) side effects include hyperglycemia, rhabdomyolysis, anorexia, neutropenia, sepsis, pneumonitis, and fatigue. Clinical responses were seen in 27% of 11 evaluable patients with one complete response (CR) in a FL patient and partial responses (PR) in two patients (DLBCL, MZL) (Table 2), and stable diseases (SD) in FL (3/4 patients), MCL (1/2 patients), and T-cell lymphoma (1/1 patients) [78]. The pattern of activity, with most of the responses in FL, is similar to what was reported with another dual PI3K/mTOR inhibitor voxtalisib (see below) [79,80].

All of the other dual PI3K/mTOR inhibitors have not reached the clinical development stage or the latter has been permanently or temporarily stopped. Among these, here we will only discuss the two agents with available clinical data, whilst we refer to the Supplementary Material for the other compounds.

**Voxtalisib** (XL765/SAR245409) preferentially targets p110γ, followed by p110α, p110δ, p110β, and TORC1/TORC2 with activity in a variety of solid tumor models [98]. It is an oral compound and passes the BBB [98]. Voxtalisib also has preclinical anti-tumor activity in models of lymphoma and CLL [103]. The positive laboratory data have led to different clinical trials as single agent and also in combination in patients with hematological cancers [79,80,104].

Sixteen patients with relapsed or refractory lymphoma (MCL, FL, DLBCL, others) received voxtalisib in an expansion cohort [79] of the phase I study for patients with solid tumors (NCT00485719) [105] (Table 2). The toxicity profile included fatigue, gastrointestinal and cutaneous adverse events, liver enzyme abnormalities, and cytopenia [79]. Three patients achieved a clinical response with one CR in a FL patients and two PR in patients (DLBCL, MCL, one patient each) [79]. Although not reaching the definition of PR, two FL experienced tumor regression and six patients had SD, with one MCL and one FL receiving treatment for four months or longer [79]. Reductions in PI3K/mTOR and ERK pathway activity were seen in the serial tumor biopsies done, which were only successfully performed in one patient (MCL with PR) [79]. There was no evident modulation of chemokine or cytokine levels by drug exposure. Only two of 12 patients that underwent serial measurements presented changes [79]. Although they were both cases with clinical responses (CR and PR), the observed changes were not concordant with no overlapping changes and with the IL16 (interleukin 16) levels behaving in the opposite way [79]. Based on these results, a phase II study with voxtalisib (NCT01403636) enrolled 167 patients with relapsed or refractory disease (FL, MCL, DLBCL, CLL) [80] (Table 2). Clinical responses were seen in all of the subtypes, but especially in FL (41%), followed by MCL and CLL (12 and 11%, respectively) [80]. Only two of the 42 (5%) DLBCL patients responded [80]. Complete responses were observed in FL (11%, 5/47) and MCL (7%, 3/42) [80]. SD was seen in 33% (14/42) of MCL patients, 30% (14/46) of FL, 10% (4/41) of DLBCL, and 66% (23/35) of CLL [80]. The median progression-free survival was 58 weeks for FL patients, 24 for CLL, nine for MCL, and seven for DLBCL [80].

No useful information came from genetic studies done on tumor samples before treatment or from the measurement of cytokines and chemokines in plasma samples in both the phase I [79] and in the phase II [80] voxtalisib studies. In the phase I study, only two of 12 patients that underwent serial measurements presented changes [79]. Although they were both cases with clinical responses (CR and PR), the observed changes were not concordant and the IL16 levels modulated in the opposite way [79].

The combination has also been studied in a phase I clinical trial study (NCT01410513), evaluating voxtalisib in combination with the anti-CD20 monoclonal antibody rituximab plus or minus the chemotherapy agent bendamustine in patients with relapsed or refractory B-cell malignancies [104]. The study enrolled 37 patients, 16 in the rituximab arm (CLL, n. = 11; MCL, n. = 3; FL, n. = 2) and 21 in the triple combination arm (CLL, n. = 12; MCL, n. = 6; FL, n. = 3) [104]. The safety profile (characterized by nausea, fatigue, and vomiting) was acceptable, with no interactions among drugs at the pharmacokinetic level [104]. There was clinical activity: one CR in FL patient and five PRs in the voxtalisib/rituximab; three CRs (one patient with MCL, two with CLL) and eight PRs in the voxtalisib/rituximab/bendamustine arm [104].

The clinical development of voxtalisib has been stopped [106] due to limited clinical anti-tumor activity observed in two phase I studies enrolling 83 (NCT00485719) [105] and 49 (NCT01596270) [106] patients with advanced solid tumors.

The LY294002/SF1101 derivative **SF1126** is a multikinase inhibitor, which targets the PI3K isoforms and TORC1/TORC2, but also DNA-PK and the BET Bromodomain proteins [107,108,109]. SF1126 has preclinical anti-tumor activity in B and T cell lymphoma models [110], as well from multiple myeloma [111]. None of the five patients with B cell lymphoid neoplasm that were enrolled in the phase I study (NCT00907205) achieved a CR or PR [81] (Table 2). Two CLL patients (one with no prior therapy) achieved a SD [81].

## 7. Future Perspective for Dual PI3K/mTOR Inhibitors

As evident from what summarized for each individual compound, dual PI3K/mTOR inhibitors that have entered the clinical evaluation have not achieved as good results as expected. The main reason could be the frequent occurrence of dose-limiting toxicities that do not allow for reaching potentially active doses [1,2,3,4,112]. This does not come as a surprise since dual PI3K/mTOR inhibitors target multiple proteins that play fundamental roles in a variety of normal tissues. The main toxicities include diarrhea [80,81,102,113,114,115,116,117], vomiting [102,114,116,117,118], nausea [80,102,114,117,118,119], rash [96,102,106,113,114,118,119,120,121], fatigue [80,102,105,106,113,114,117,118,121,122], decreased appetite [80,105,117,118,119], hyperglycemia [96,102,114,115,119,120,122,123], mucositis [89,96,114,115,118,122], increase in liver enzymes [96,102,105,124], and thrombocytopenia [114,122,124]. The additional dose limiting toxicities reported in individual trials, which does mean they are specific to a single compound, include allergic reactions [124], bradycardia and myocardial ischemia [89], dysgeusia [118], hypertension [102], pneumonitis [115], uveitis [114], and suicide attempt [102].

Different dosing schedules could improve the toxicity profile and provide the best therapeutic window. For example, apitolisib given once weekly [123] has shown better tolerability than once daily [120]. Other approaches include intermittent dosing, dose reductions, and interruptions that are based on side effects [1,2,3].

Alternative modalities to decrease the side effects that have been explored are the delivery of the compounds preferentially to cancer cells (sparse preclinical data with BEZ235 in CaCO(3) nanocrystals [125] or in liposomes [126]) or lowering the doses of the dual PI3K/mTOR inhibitors in the context of combination therapies can help to overcome the toxicity issue. Combinations would also be important for two additional reasons. First, the addition of a second compound can overcome the primary or acquired resistance to the dual PI3K/mTOR inhibitors [3,4,5]. Indeed, resistance to both PI3K and mTOR inhibitors is due to genetic events that maintain the same pathway active or via activation of alternative signaling cascades [3,4,64]. Second, the activation of PI3K/mTOR signaling is a frequent mechanism of resistance to other targeted agents [3,4]. Studies conducted mainly in the solid tumor setting suggest that combinations with other agents can be feasible [104,113,114,127,128], although toxicities can still be an issue [106,127,129,130,131,132,133].

Preclinical data that were obtained in different lymphoma models indicate that dual PI3K/mTOR inhibitors synergize with both targeted and chemotherapy agents, as presented in Table 4. For few combinations, synergism has been observed in different laboratories and/or obtained with different dual PI3K/mTOR inhibitors, underlying the robustness of the data.

While also considering the toxicity profile and its already established role in CLL and lymphomas [151], the BCL2 inhibitor venetoclax appears as an interesting drug to be combined with dual PI3K/mTOR inhibitors. Bimiralisib, dactolisib, and omipalisib have all shown synergism when combined with venetoclax [75,139,140,141]. Combining dactolisib with venetoclax induces the accumulation of pro-apoptotic BAD and BIM and down-regulation of the anti-apoptotic MCL1 [139,140], with the ability to revert secondary resistance to venetoclax [139]. The combination of bimiralisib with venetoclax is much more active than the single agents in GCB and ABC DLBCL xenograft models, as well as causing an increase in cell death in CLL primary cells [75].

Another combination that is sustained by multiple data is with histone deacetylase (HDAC) inhibitors. Dactolisib, bimiralisib, and omipalisib have been successfully combined with panobinostat [75,145] or Vorinostat [141]. In ABC, GCB and double hit DLBCL, and MCL cell lines, dactolisib had major effect when combined with HDAC inhibitor panobinostat, AKT inactivation, MCL1 downregulation, and BIM upregulation contribute to the effect of the combination of dactolisib with panobinostat [145,147,152].

Combination with the FDA approved BTK inhibitor ibrutinib appears to be another interesting combination, as shown using apitolisib, bimiralisib [75], and dactolisib [143] in ABC DLBCL and MCL models [75,143].

Bimiralisib and other BCR signaling inhibitors induce the increased expression of both PIM1 and PIM2, kinases that are involved in lymphomagenesis and potential therapeutic targets. The combination of bimiralisib with the PIM inhibitor AZD1208 [153] is largely synergistic in both GCB and ABC DLBCL cells with an increased G0/G1 cell cycle arrest [75]. The addition of the PIM inhibitor SGI-1776 to dactolisib is associated with MCL1 downregulation and increased cell death in the ABC DLBCL cell lines [76].

Bimiralisib [75] and PF04691502 [136] have been beneficially combined with the anti-CD20 monoclonal antibody rituximab in the DLBCL and MCL cell lines. The addition of rituximab to a dual PI3K/mTOR inhibitors with rituximab is the only combination that has been clinically evaluated. Indeed, we have already mentioned the voxtalisib phase I trial that has reported some clinical activity [104].

Similar results have been obtained with three dual PI3K/mTOR inhibitors (dactolisib, bimiralisib, and omipalisib) added to proteasome inhibitors (bortezomib or marizomib) [75,141,144,149]. In particular, the addition of dactolisib reverts the resistance to proteasome inhibitors in a bortezomib-resistant MCL cell line decreasing AKT and mTOR signaling [144].

Finally, the effect of this class of agents on the tumor microenvironment and how this indirect activity can be exploited, especially in combination with immuno-oncology drugs, such as the bispecific antibodies, is an open issue [154,155].

## 8. Conclusions

Based on the importance of the p110α in different solid tumors, a bigger relevance to this isoform has been given in the design of compounds and in the population of patients that are enrolled in the clinical studies. However, there are plenty of preclinical data sustaining the anti-tumor activity of dual PI3K/mTOR inhibitors as single agents and in combination in lymphomas. Additionally, clinical responses, including CR (especially in the FL setting), are clearly observed in the very few clinical studies that were performed in patients affected by relapsed/refractory lymphomas or CLL. Unfortunately, the vast majority of clinical trials that were performed in patients with solid tumors have been disappointing due to unacceptable toxicity profile and/or to a lack of meaningful clinical activity. Based on these clinical results, the clinical development of all but three dual PI3K/mTOR inhibitors has been stopped, although they could be beneficial for some patients that were affected by lymphoid neoplasms. Different schedules of dual PI3K/mTOR inhibitors given as single agents in specific patients’ populations (for example, high risk or relapsed/refractory FL patients) or combination regimens (for example, with venetoclax) appear still worthy of further clinical investigations. The use of tools to identify the responders at an early time-point, possibly paired with still-to-be defined robust biomarkers, will help in optimizing the use of dual PI3K/mTOR inhibitors, avoiding both toxicities to patients that are unlikely to benefit and costs to the health care system.

## Figures and Tables

**Table 1:** List of dual Pi3K/mTOR inhibitors sorted by their official name, if assigned, or by their common/alternative name.

Official Name	Common/Alternative Name	Company/Developer	Ability to Cross the BBB	Clinical Stage	Orphan Drug Status	Development Status *
Apitolisib	GDC-0980, RG7422	Genentech; Piramed	No/low	Phase I/II	-	Discontinued *
Bimiralisib	PQR309	Piqur Therapeutics	yes	Phase I/II	DLBCL	On-going trials
Dactolisib	BEZ235, NVP-BEZ235, RTB-101, NVP-BEZ235-NX	Novartis; resTORbio	n.a.			
Gedatolisib	PF-05212384/PKI-587, 1197160-78-3	Wyeth; Pfizer	n.a.	Phase I/II/III	-	On-going trials
Omipalisib	GSK2126458, GSK458, GSK-212	GlaxoSmithKline	yes	Phase I	-	No on-ongoing trials
Panulisib	P7170, S9WA04F921	Piramal Healthcare	n.a.	Phase I	-	No on-going trials
Samotolisib	LY3023414, GTPL8918	Eli Lilly and Company	n.a.	Phase I/II	-	Discontinued *
Voxtalisib	XL765, SAR245409	EMD Serono; Exelixis; Sanofi	yes	Phase I/II	-	Discontinued *
-	BGT226, NVP-BGT226	Novartis	n.a.	Phase I/II	-	Discontinued *
-	DS7423, 70895382	Daiichi Sankyo	yes	Phase I	-	Discontinued *
-	GDC-0084, RG 7666	Genentech; Kazia Therapeutics	yes	Phase I/II/III	glioblastoma multiforme	On-going trials
-	GNE-477	Genentech	n.a.	-	-	No on-going trials
-	PF-04691502	Pfizer	n.a.	Phase I/II	-	Discontinued *
-	PF-04979064	Pfizer	n.a.	-	-	No on-going trials
-	PI-103, 9884685	Merck	yes	-	-	No on-going trials
-	PKI-179	Wyeth; Pfizer	n.a.	-	-	Discontinued *
-	PKI-402, 44187953	Wyeth	n.a.	-	-	No on-going trials
-	PQR530	Piqur Therapeutics	yes	-	-	No on-going trials
-	PWT33597, VDC-597	Pathway Therapeutics; VetDC	n.a.	Phase I	-	No on-going trials
-	SF-1126	Semafore; SignalRx Pharmaceuticals	n.a.	Phase I	CLL	Status unknown
-	SN32976, 1246202-11-8	The University of Auckland	n.a.	-	-	Status unknown
-	VS-5584, SB2343	S*BIO; Verastem	n.a.	Phase I	Mesothelioma	Discontinued *

n.a., data not available; *, based on http://adisinsight.springer.com/ and/or https://clinicaltrials.gov accessed in December 2019. BBB, blood brain barrier; DLBCL, diffuse large B cell lymphoma; CLL, chronic lymphocytic leukemia.

**Table 2:** Clinical trials evaluating dual PI3K/mTOR inhibitors as single agents that have enrolled lymphoma patients *.

Drug	Phase	Trial	Lymphoma Population	Overall Response Rate	Complete Remission Rate	Partial Response Rate
Bimiralisib	I/II	NCT02249429 [78]^	53, R/R	Whole cohort, 27% (3/11) ^ DLBCL 100% (1/1) FL, 25% (1/4) T-cell lymphoma, 0% (0/1) MZL, 100% (1/1) MCL, 0% (0/2) HL, 0% (0/2)	Whole cohort, 9% (1/11) ^ DLBCL, 0% (0/1) FL, 25% (1/4) T-cell lymphoma, 0% (0/1) MZL, 0% (0/1) MCL, 0% (0/2) HL, 0% (0/2)	Whole cohort, 18% (2/11) ^ DLBCL 100% (1/1) FL, 0% (0/4) T-cell lymphoma, 0% (0/1) MZL, 100% (1/1) MCL, 0% (0/2) HL, 0% (0/2)
Bimiralisib	II	NCT03127020	9, R/R	n.r.	n.r.	n.r.
Bimiralisib	II	NCT02669511	21, R/R PCNSL	n.r.	n.r.	n.r.
Voxtalisib	I	NCT00485719 [79]	16 **, R/R	Whole cohort, 19% (3/16) FL, 20% (1/5) MCL, 17% (1/6) DLBCL, 50% (1/2)	Whole cohort, 6% (1/16) FL, 20% (1/5) MCL, 0% (0/6) DLBCL, 0% (0/2)	Whole cohort, 13% (2/16) FL, 0% (0/5) MCL, 17% (1/6) DLBCL, 50% (1/2)
Voxtalisib	II	NCT01403636 [80]	167 ***, R/R	Whole cohort, 18% (30/167); FL, 41% (19/47); MCL, 12% (5/42); CLL, 11% (4/36); DLBCL, 5% (2/42).	Whole cohort, 5%; FL, 11% (5/47); MCL, 7% (3/42); CLL, 0% (0/36); DLBCL, 0% (0/42).	Whole cohort, 13%; FL, 30% (14/47); MCL, 5% (2/42); CLL, 11% (4/36); DLBCL, 5% (2/42).
SF1126	I ^^	NCT00907205 [81]	5 ****, R/R	Whole cohort, 0% (0/16) CLL, 0% (0/4) DLBCL 0% (0/1)	Whole cohort, 0% (0/16) CLL, 0% (0/4) DLBCL 0% (0/1)	Whole cohort, 0% (0/16) CLL, 0% (0/4) DLBCL 0% (0/1)

*, based on what reported in https://clinicaltrials.gov accessed in December 2019; **, 6 MCL, 5 FL, 2 DLBCL, 1 anaplastic large cell lymphoma, 1 HL, 1 transformed [79]; ***, 47 FL, 42 MCL, 42 DLBCL, 36 CLL [80]; ****, 1 DLBCL, 4 CLL [82]; n.r., not reported; ^, based on 11 evaluable patients reported in an abstract [78]; ^^, the trial also allowed the addition of rituximab.

**Table 3:** Chemical structures of the three dual PI3K/mTOR inhibitors still in clinical development. Data collected from https://www.ebi.ac.uk/chembl/ [83], http://zinc.docking.org/substances/home/ [84], https://pubchem.ncbi.nlm.nih.gov/ [85], https://www.drugbank.ca/ [86], https://fdasis.nlm.nih.gov/srs/. MW, molecular weight. IUPAC, International Union of Pure and Applied Chemistry. Additional dual PI3K/mTOR inhibitors are presented in Table S1.

Official /Common/ Alternative Name	3D-Structure	IUPAC Name	MW
Bimiralisib, PQR309	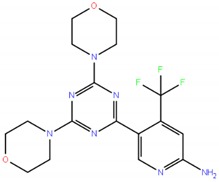	5-[4,6-bis(morpholin-4-yl)-1,3,5-triazin-2-yl]-4-(trifluoromethyl)pyridin-2-amine	411.39
Gedatolisib, PF-05212384/ PKI-587, 1197160-78-3	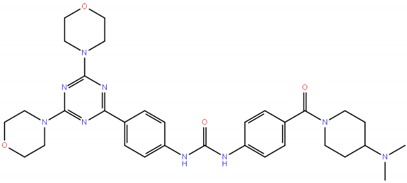	1-{4-[4,6-bis(morpholin-4-yl)-1,3,5-triazin-2-yl]phenyl}-3-{4-[4-(dimethylamino)piperidine-1-carbonyl]phenyl}urea	615.74
GDC-0084, RG 7666	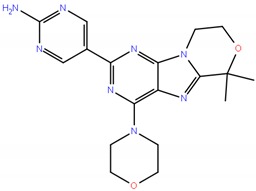	5-[6,6-dimethyl-4-(morpholin-4-yl)-6H,8H,9H-[1,4]oxazino [3,4-h]purin-2-yl]pyrimidin-2-amine	382.4

**Table 4:** Combinations based on dual PI3K/mTOR inhibitors with available preclinical data in lymphomas.

Additional Mechanism of Action	Combination Partner	PI3K/mTOR Inhibitor	Disease Model
AKT inhibition	Perifosine [134], Oridonin [135]	Dactolisib [134]	ABC DLBCL [135], MCL [134]
Anti-CD20 monoclonal antibody	Rituximab	Bimiralisib [75], PF04691502 [136]	DLBCL [75,136], MCL [136]
Anti-CD30 antibody drug conjugate	Brentuximab vedotin	Omipalisib, BGT226 [137],	HL [137]
Autophagy inhibition	Chloroquine	Dactolisib [138]	GCB DLBCL, MCL, T-NHL [138]
BCL2 inhibition	Venetoclax	Bimiralisib [75], Dactolisib [139,140], Omipalisib [141]	ABC DLBCL [75], GCB DLBCL [75,139,140], MCL [75], CLL [75], T-NHL [141]
BCL2/BCL-XL inhibition	Navitoclax	Dactolisib [76]	GCB DLBCL [76]
BCL2/BCL-XL/MCL1 inhibition	Obatoclax	Dactolisib [76]	ABC DLBCL [76]
BET Bromodomain degradation	ARV-825	Bimiralisib [75]	DLBCL [75]
BET Bromodomain inhibition	JQ1	Dactolisib [142]	Murine T-NHL [142]
BTK inhibition	Ibrutinib	Apitolisib [143], Bimiralisib [75], Dactolisib [143]	ABC DLBCL [75,143], MCL [75]
Chemotherapy	Doxorubicin	Dactolisib [144], Omipalisib [141]	MCL [144], T-NHL [141]
Chemotherapy	Vincristine	Dactolisib [142]	MCL, murine T-NHL [142]
Complex I (NADPH:ubiquinone oxidoreductase) inhibition	Metformin	Bimiralisib [75]	DLBCL [75]
HDAC inhibition	Panobinostat [75,145], Vorinostat [141]	Dactolisib [145], Bimiralisib [75], Omipalisib [141]	DLBCL [75,145], MCL [145], CLL [75], T-NHL [141]
IRF4/SPIB inhibition	Lenalidomide	Bimiralisib [75], Dactolisib [146]	ABC DLBCL [75,146]
JAK1/2 inhibition	INCB16562	Dactolisib [147]	DLBCL [147]
MEK inhibition	AZD6244	Dactolisib [148]	GCB DLBCL, BL [148]
mTOR inhibition	Everolimus	Dactolisib [134]	MCL [134]
Multikinase inhibition	Enzastaurin	Dactolisib [134]	MCL [134]
Myc inhibition	10058-F4	Dactolisib [142]	Murine T-NHL [142]
NF-κB inhibition	BAY-11-7082	Dactolisib [76]	ABC-DLBCL [76]
PAK1 inhibition	IPA-3	Dactolisib [74]	DLBCL [74]
PIM inhibition	SGI-1776 [76], AZD1208 [75]	Dactolisib [76], Bimiralisib [75]	ABC DLBCL [75,76], GCB-DLBCL [75]
Proteasome inhibition	Bortezomib [144,149], [141], Marizomib [75]	Dactolisib [144,149], Bimiralisib [75], Omipalisib [141]	ABC DLBCL [75], t-FL/GCB DLBCL [149], MCL [144], T-NHL [141].
Steroids	Dexamethasone [150]	Omipalisib [150]	T-NHL [150]

ABC DLBCL, activated B-cell like diffuse large B cell lymphoma; GCB DLBCL, germinal center B-cell type diffuse large B cell lymphoma; MCL, mantle cell lymphoma; t-FL, transformed follicular lymphoma; CLL, chronic lymphocytic leukemia; HL, Hodgkin lymphoma; T-NHL: T-cell lymphoma.

## Supplementary Materials

Supplementary materials can be found at https://www.mdpi.com/1422-0067/21/3/1060/s1.
