# Supplementary material for: Is There a Role for Dual PI3K/mTOR Inhibitors for Patients Affected with Lymphoma?
Source: Int J Mol Sci. 2020 Feb 5;21(3):1060. doi: 10.3390/ijms21031060 (PMC7037719; doi:10.3390/ijms21031060)
Supplement: Supplementary file 1 [file ijms-21-01060-s001.pdf]

Is there a role for dual PI3K/mTOR inhibitors for patients affected with lymphoma?

Chiara Tarantelli [1], Antonio Lupia [2], Anastasios Stathis [3,4], Francesco Bertoni [1,3]
[1] Institute of Oncology Research, Faculty of Biomedical Sciences, USI, Bellinzona, Switzerland; [2] Department of Health Sciences, University "Magna Græcia" of Catanzaro, Catanzaro, Italy; [3], Oncology Institute of Southern Switzerland, Bellinzona, Switzerland; [4] Faculty of Biomedical Sciences, USI, Lugano, Switzerland.

Supplementary Material

Additional Dual PI3K/mTOR inhibitors

We discuss here the dual PI3K/mTOR inhibitors with no available clinical data in patients with lymphoma.

Dactolisib (BEZ235/NVP-BEZ235) is the most studied dual PI3K/mTOR inhibitor. It is a potent inhibitor of all the four PI3K isoforms (slightly less for the p110β), of TORC1/TORC2 and also of the PI3Kα mutants most commonly detected in solid tumors [1]. Dactolisib has preclinical anti-tumor activity in different models of B and T cell lymphomas [2-18]. The compound can also act as microtubule destabilizer [7] and it inhibits DNA damage response (DDR) kinases [12]. Indeed, Shortt and Coll. suggested that it is indeed the triple inhibition of PI3K, mTOR and DDR kinases playing a fundamental role in the higher activity seen with dactolisib in inducing apoptosis in Myc-driven mouse lymphoma models in comparison with single PI3K or mTOR inhibitors [12]. The clinical program for dactolisib has been stopped due to a high variable pharmacokinetic profile observed in the phase I studies [19]. Despite all the preclinical data available, to the best of our knowledge, no lymphoma patient has been treated with dactolisib.

BGT226/NVP-BGT226 inhibits p110α, p110β, p110δ, and p110γ, with a preference for p110α (wild type and mutated), and TORC1/TORC2 [20]. Besides showing activity in solid tumors, acute leukemia and multiple myeloma models [20-23], the compound has shown preclinical anti-lymphoma activity [16, 24]. Without enrolling any lymphoma patient, a phase I study performed in USA and Europe led to a stop in the clinical development of BGT226 due to the inability of reaching concentrations expected to properly inhibit PI3K signaling in the absence of dose-limiting toxicity [25]. Similar toxicity was observed in a parallel phase I study performed in Japanese patients with solid tumors, which stopped before reaching the dose-limiting toxicity based on the already available of the other study [26]. Resistance to BEZ235 and BGT226 is associated to higher expression of PAK1 gene, and combination of PI3K inhibition and PAK1 inhibition is synergistic [16].

PI103 is a strong inhibitor of p110α, p110β, p110δ L, p110γ, TORC1/TORC2 and also of DNA-PK with preclinical anti-tumor activity in solid tumors [27, 28] and mouse and human T-cell lymphoma models [29-31].

Apitolisib (GDC0980/RG7422/apitolisib) is a potent inhibitor of all the PI3K and of TORC1/TORC2 with anti-tumor activity in different solid tumor cell lines [32, 33]. Apitolisib has also strong *in vitro* activity in B cell lymphoma cell lines [34]. No patient with lymphoma was enrolled in the phase I (NCT00854152) [35], and no information on recruited patients is available from a second study (NCT00854126) [36], already closed.

PF04691502 inhibits the four PI3K isoforms, including mutant p110α, and TORC1/TORC2 and has preclinical activity in solid tumors [37], DLBCL, MCL [38] and cutaneous T cell lymphomas [39]. No study has been performed for patients with lymphoma.

Omipalisib (GSK2126458/GSK458) is an inhibitor of p110α (wild type and mutant), p110β, p110δ, p110γ, TORC1 and TORC2 with activity in cell lines derived from solid tumors [40], from BL [41] and T cell lymphomas [42]. Omipalisib has not been clinically evaluated in patients with lymphoma.

VS5584/SB2343 has shown activity in solid tumor, BL [43, 44] and MM cell lines [43]. No data are available of the phase I study (NCT01991938) that had already enrolled 75 patients and was terminated due to lack of recruitment and the company's decision to de-prioritize compound development.

Finally, **NU7441** is believed to mainly act as a DNA-PK inhibitor but it also targets PI3Ks and mTOR [45]. It has preclinical activity in cell lines derived from BL and from other lymphoma subtypes [46].

To the best of our knowledge, there are no reported data of activity in lymphoma models using **DS7423** [47], the GDC-0941 derivative **GNE-477** [48], the PF-04691502 derivative **PF-04979064** [49], **PKI-179** [50], **PKI-402** [51, 52], **PQR530** [53], **PWT33597** (VDC-597 currently commercialized as veterinary anticancer drug) [54], **samotolisib** (LY3023414) [55, 56], **SN32976** [57, 58], **WJD008** [59], and for the multitarget PI3K/mTOR/ALK-1/DNA-PK inhibitor **Panulisib** (P7170) [60-62].

References

1. Maira, S. M.; Stauffer, F.; Brueggen, J.; Furet, P.; Schnell, C.; Fritsch, C.; Brachmann, S.; Chene, P.; De Pover, A.; Schoemaker, K.; Fabbro, D.; Gabriel, D.; Simonen, M.; Murphy, L.; Finan, P.; Sellers, W.; Garcia-Echeverria, C., Identification and characterization of NVP-BEZ235, a new orally available dual phosphatidylinositol 3-kinase/mammalian target of rapamycin inhibitor with potent in vivo antitumor activity. *Mol Cancer Ther* **2008,** 7, (7), 1851-63.
2. Aust, M.; Wallace, L.; Grant, S., Inhibition Of PI3K/mTOR By BEZ235 Dramatically Potentiates Panobinostat-Induced Lethality In Diffuse Large B-Cell Lymphoma Through Multiple Mechanisms. *Blood* **2013,** 122, (21), 817-817.
3. Rahmani, M.; Aust, M. M.; Benson, E. C.; Wallace, L.; Friedberg, J.; Grant, S., PI3K/mTOR inhibition markedly potentiates HDAC inhibitor activity in NHL cells through BIM- and MCL-1-dependent mechanisms in vitro and in vivo. *Clinical cancer research : an official journal of the American Association for Cancer Research* **2014,** 20, (18), 4849-60.
4. Buglio, D.; Lemoine, M.; Neelapu, S. S.; Vega, F.; Berry, D.; Younes, A., NVP-BEZ235, A Dual Inhibitor of Phosphoinositol-3-Kinase (PI3K) and Mammalian Target of Rapamycin (mTOR), Is a Potent Inhibitor of Lymphoma Cell Growth and Survival. *Blood* **2011,** 118, (21), 4965-4965.
5. Bhende, P. M.; Park, S. I.; Lim, M. S.; Dittmer, D. P.; Damania, B., The dual PI3K/mTOR inhibitor, NVP-BEZ235, is efficacious against follicular lymphoma. *Leukemia* **2010,** 24, (10), 1781-4.
6. Kim, A.; Park, S.; Lee, J. E.; Jang, W. S.; Lee, S. J.; Kang, H. J.; Lee, S. S., The dual PI3K and mTOR inhibitor NVP-BEZ235 exhibits anti-proliferative activity and overcomes bortezomib resistance in mantle cell lymphoma cells. *Leukemia research* **2012,** 36, (7), 912-20.
7. Civallero, M.; Cosenza, M.; Pozzi, S.; Bari, A.; Ferri, P.; Sacchi, S., Activity of BKM120 and BEZ235 against Lymphoma Cells. *BioMed Research International* **2015,** 2015, 870918.
8. Anders, P.; Bhende, P. M.; Foote, M.; Dittmer, D. P.; Park, S. I.; Damania, B., Dual inhibition of phosphatidylinositol 3-kinase/mammalian target of rapamycin and mitogen activated protein kinase pathways in non-Hodgkin lymphoma. *Leukemia & lymphoma* **2015,** 56, (1), 263-6.
9. Vergaro, V.; Civallero, M.; Citti, C.; Cosenza, M.; Baldassarre, F.; Cannazza, G.; Pozzi, S.; Sacchi, S.; Fanizzi, F. P.; Ciccarella, G., Cell-Penetrating CaCO(3) Nanocrystals for Improved Transport of NVP-BEZ235 across Membrane Barrier in T-Cell Lymphoma. *Cancers (Basel)* **2018,** 10, (2), 31.
10. Choudhary, G. S.; Al-Harbi, S.; Mazumder, S.; Hill, B. T.; Smith, M. R.; Bodo, J.; Hsi, E. D.; Almasan, A., MCL-1 and BCL-xL-dependent resistance to the BCL-2 inhibitor ABT-199 can be overcome by preventing PI3K/AKT/mTOR activation in lymphoid malignancies. *Cell Death Dis* **2015,** 6, (e1593), e1593.
11. Civallero, M.; Cosenza, M.; Marcheselli, L.; Pozzi, S.; Sacchi, S., NVP-BEZ235 alone and in combination in mantle cell lymphoma: an effective therapeutic strategy. *Expert opinion on investigational drugs* **2012,** 21, (11), 1597-606.
12. Shortt, J.; Martin, B. P.; Newbold, A.; Hannan, K. M.; Devlin, J. R.; Baker, A. J.; Ralli, R.; Cullinane, C.; Schmitt, C. A.; Reimann, M.; Hall, M. N.; Wall, M.; Hannan, R. D.; Pearson, R. B.; McArthur, G. A.; Johnstone, R. W., Combined inhibition of PI3K-related DNA damage response kinases and mTORC1 induces apoptosis in MYC-driven B-cell lymphomas. *Blood* **2013,** 121, (15), 2964-74.
13. Bhatt, A. P.; Bhende, P. M.; Sin, S. H.; Roy, D.; Dittmer, D. P.; Damania, B., Dual inhibition of PI3K and mTOR inhibits autocrine and paracrine proliferative loops in PI3K/Akt/mTOR-addicted lymphomas. *Blood* **2010,** 115, (22), 4455-63.
14. Rosich, L.; Montraveta, A.; Xargay-Torrent, S.; López-Guerra, M.; Roldán, J.; Aymerich, M.; Salaverria, I.; Beà, S.; Campo, E.; Pérez-Galán, P.; Roué, G.; Colomer, D., Dual PI3K/mTOR inhibition is required to effectively impair microenvironment survival signals in mantle cell lymphoma. *Oncotarget* **2014,** 5, (16).
15. Furukawa, S.; Wei, L.; Krams, S. M.; Esquivel, C. O.; Martinez, O. M., PI3Kdelta inhibition augments the efficacy of rapamycin in suppressing proliferation of Epstein-Barr virus (EBV)+ B cell lymphomas. *Am J Transplant* **2013,** 13, (8), 2035-43.
16. Walsh, K.; McKinney, M. S.; Love, C.; Liu, Q.; Fan, A.; Patel, A.; Smith, J.; Beaven, A.; Jima, D. D.; Dave, S. S., PAK1 mediates resistance to PI3K inhibition in lymphomas. *Clin Cancer Res* **2013,** 19, (5), 1106-15.
17. Li, C.; Xin, P.; Xiao, H.; Zheng, Y.; Huang, Y.; Zhu, X., The dual PI3K/mTOR inhibitor NVP-BEZ235 inhibits proliferation and induces apoptosis of burkitt lymphoma cells. *Cancer Cell International* **2015,** 15, (1), 65.

18. Zang, C.; Eucker, J.; Liu, H.; Muller, A.; Possinger, K.; Scholz, C. W., Concurrent inhibition of PI3-kinase and mTOR induces cell death in diffuse large B cell lymphomas, a mechanism involving down regulation of Mcl-1. *Cancer letters* **2013,** 339, (2), 288-97.
19. Rodon, J.; Perez-Fidalgo, A.; Krop, I. E.; Burris, H.; Guerrero-Zotano, A.; Britten, C. D.; Becerra, C.; Schellens, J.; Richards, D. A.; Schuler, M.; Abu-Khalaf, M.; Johnson, F. M.; Ranson, M.; Edenfield, J.; Silva, A. P.; Hackl, W.; Quadt, C.; Demanse, D.; Duval, V.; Baselga, J., Phase 1/1b dose escalation and expansion study of BEZ235, a dual PI3K/mTOR inhibitor, in patients with advanced solid tumors including patients with advanced breast cancer. *Cancer Chemother Pharmacol* **2018,** 82, (2), 285-298.
20. Chang, K. Y.; Tsai, S. Y.; Wu, C. M.; Yen, C. J.; Chuang, B. F.; Chang, J. Y., Novel phosphoinositide 3-kinase/mTOR dual inhibitor, NVP-BGT226, displays potent growth-inhibitory activity against human head and neck cancer cells in vitro and in vivo. *Clin Cancer Res* **2011,** 17, (22), 7116-26.
21. Kampa-Schittenhelm, K. M.; Heinrich, M. C.; Akmut, F.; Rasp, K. H.; Illing, B.; Dohner, H.; Dohner, K.; Schittenhelm, M. M., Cell cycle-dependent activity of the novel dual PI3K-MTORC1/2 inhibitor NVP-BGT226 in acute leukemia. *Mol Cancer* **2013,** 12, 46.
22. Baumann, P.; Schneider, L.; Mandl-Weber, S.; Oduncu, F.; Schmidmaier, R., Simultaneous targeting of PI3K and mTOR with NVP-BGT226 is highly effective in multiple myeloma. *Anticancer Drugs* **2012,** 23, (1), 131-8.
23. Katanasaka, Y.; Kodera, Y.; Yunokawa, M.; Kitamura, Y.; Tamura, T.; Koizumi, F., Synergistic anti-tumor effects of a novel phosphatidyl inositol-3 kinase/mammalian target of rapamycin dual inhibitor BGT226 and gefitinib in non-small cell lung cancer cell lines. *Cancer Lett* **2014,** 347, (2), 196-203.
24. Graf, N.; Li, Z.; Herrmann, K.; Weh, D.; Aichler, M.; Slawska, J.; Walch, A.; Peschel, C.; Schwaiger, M.; Buck, A. K.; Dechow, T.; Keller, U., Positron emission tomographic monitoring of dual phosphatidylinositol-3-kinase and mTOR inhibition in anaplastic large cell lymphoma. *OncoTargets and therapy* **2014,** 7, 789-98.
25. Markman, B.; Tabernero, J.; Krop, I.; Shapiro, G. I.; Siu, L.; Chen, L. C.; Mita, M.; Melendez Cuero, M.; Stutvoet, S.; Birle, D.; Anak, O.; Hackl, W.; Baselga, J., Phase I safety, pharmacokinetic, and pharmacodynamic study of the oral phosphatidylinositol-3-kinase and mTOR inhibitor BGT226 in patients with advanced solid tumors. *Ann Oncol* **2012,** 23, (9), 2399-408.
26. Minami, H.; Fujiwara, Y.; Muro, K.; Sato, M.; Moriya, A., Phase I study of BGT226, a pan-PI3K and mTOR inhibitor, in Japanese patients with advanced solid cancers. *Cancer Chemother Pharmacol* **2019,** 84, (2), 337-343.
27. Raynaud, F. I.; Eccles, S.; Clarke, P. A.; Hayes, A.; Nutley, B.; Alix, S.; Henley, A.; Di-Stefano, F.; Ahmad, Z.; Guillard, S.; Bjerke, L. M.; Kelland, L.; Valenti, M.; Patterson, L.; Gowan, S.; de Haven Brandon, A.; Hayakawa, M.; Kaizawa, H.; Koizumi, T.; Ohishi, T.; Patel, S.; Saghir, N.; Parker, P.; Waterfield, M.; Workman, P., Pharmacologic characterization of a potent inhibitor of class I phosphatidylinositide 3-kinases. *Cancer research* **2007,** 67, (12), 5840-50.
28. Bagci-Onder, T.; Wakimoto, H.; Anderegg, M.; Cameron, C.; Shah, K., A dual PI3K/mTOR inhibitor, PI-103, cooperates with stem cell-delivered TRAIL in experimental glioma models. *Cancer research* **2011,** 71, (1), 154-63.
29. Maurya, A. K.; Vinayak, M., PI-103 attenuates PI3K-AKT signaling and induces apoptosis in murineT-cell lymphoma. *Leukemia & lymphoma* **2017,** 58, (5), 1153-1161.
30. Shepherd, C.; Banerjee, L.; Cheung, C. W.; Mansour, M. R.; Jenkinson, S.; Gale, R. E.; Khwaja, A., PI3K/mTOR inhibition upregulates NOTCH-MYC signalling leading to an impaired cytotoxic response. *Leukemia* **2013,** 27, (3), 650-60.
31. Chiarini, F.; Fala, F.; Tazzari, P. L.; Ricci, F.; Astolfi, A.; Pession, A.; Pagliaro, P.; McCubrey, J. A.; Martelli, A. M., Dual inhibition of class IA phosphatidylinositol 3-kinase and mammalian target of rapamycin as a new therapeutic option for T-cell acute lymphoblastic leukemia. *Cancer research* **2009,** 69, (8), 3520-8.
32. Sutherlin, D. P.; Bao, L.; Berry, M.; Castanedo, G.; Chuckowree, I.; Dotson, J.; Folks, A.; Friedman, L.; Goldsmith, R.; Gunzner, J.; Heffron, T.; Lesnick, J.; Lewis, C.; Mathieu, S.; Murray, J.; Nonomiya, J.; Pang, J.; Pegg, N.; Prior, W. W.; Rouge, L.; Salphati, L.; Sampath, D.; Tian, Q.; Tsui, V.; Wan, N. C.; Wang, S.; Wei, B.; Wiesmann, C.; Wu, P.; Zhu, B. Y.; Olivero, A., Discovery of a potent, selective, and orally available class I phosphatidylinositol 3-kinase (PI3K)/mammalian target of rapamycin (mTOR) kinase inhibitor (GDC-0980) for the treatment of cancer. *J Med Chem* **2011,** 54, (21), 7579-87.
33. Wallin, J. J.; Edgar, K. A.; Guan, J.; Berry, M.; Prior, W. W.; Lee, L.; Lesnick, J. D.; Lewis, C.; Nonomiya, J.; Pang, J.; Salphati, L.; Olivero, A. G.; Sutherlin, D. P.; O'Brien, C.; Spoerke, J. M.; Patel, S.; Lensun, L.; Kassees, R.; Ross, L.; Lackner, M. R.; Sampath, D.; Belvin, M.; Friedman, L. S., GDC-0980 is a novel class I PI3K/mTOR kinase inhibitor with robust activity in cancer models driven by the PI3K pathway. *Molecular cancer therapeutics* **2011,** 10, (12), 2426-36.
34. Tarantelli, C.; Gaudio, E.; Arribas, A. J.; Kwee, I.; Hillmann, P.; Rinaldi, A.; Cascione, L.; Spriano, F.; Bernasconi, E.; Guidetti, F.; Carrassa, L.; Pittau, R. B.; Beaufils, F.; Ritschard, R.; Rageot, D.; Sele, A.; Dossena, B.; Rossi, F. M.; Zucchetto, A.; Taborelli, M.; Gattei, V.; Rossi, D.; Stathis, A.; Stussi, G.; Broggini, M.; Wymann, M. P.; Wicki, A.; Zucca, E.; Cmiljanovic, V.; Fabbro, D.; Bertoni, F., PQR309 Is a Novel Dual PI3K/mTOR Inhibitor with Preclinical Antitumor Activity in Lymphomas as a Single Agent and in Combination Therapy. *Clin Cancer Res* **2018,** 24, (1), 120-129.

35. Dolly, S. O.; Wagner, A. J.; Bendell, J. C.; Kindler, H. L.; Krug, L. M.; Seiwert, T. Y.; Zauderer, M. G.; Lolkema, M. P.; Apt, D.; Yeh, R. F.; Fredrickson, J. O.; Spoerke, J. M.; Koeppen, H.; Ware, J. A.; Lauchle, J. O.; Burris, H. A., 3rd; de Bono, J. S., Phase I Study of Apitolisib (GDC-0980), Dual Phosphatidylinositol-3-Kinase and Mammalian Target of Rapamycin Kinase Inhibitor, in Patients with Advanced Solid Tumors. *Clinical cancer research : an official journal of the American Association for Cancer Research* **2016,** 22, (12), 2874-84.
36. Hollebecque, A.; Clamp, A.; Horsley, L.; Morgan, J. A.; Bahleda, R.; George, S.; Shaw, D.; Lauchle, J. O.; Ware, J.; Desai, R.; Wu, J.; Fu, L.; Jayson, G. C.; Soria, J.-C.; Wagner, A. J., Abstract B153: A phase I study evaluating the pharmacokinetics (PK) and pharmacodynamic (PD) activity of the dual PI3K/mTOR inhibitor GDC-0980 administered once weekly (QW). *Molecular Cancer Therapeutics* **2011,** 10, (11 Supplement), B153-B153.
37. Yuan, J.; Mehta, P. P.; Yin, M.-J.; Sun, S.; Zou, A.; Chen, J.; Rafidi, K.; Feng, Z.; Nickel, J.; Engebretsen, J.; Hallin, J.; Blasina, A.; Zhang, E.; Nguyen, L.; Sun, M.; Vogt, P. K.; McHarg, A.; Cheng, H.; Christensen, J. G.; Kan, J. L. C.; Bagrodia, S., PF-04691502, a Potent and Selective Oral Inhibitor of PI3K and mTOR Kinases with Antitumor Activity. *Molecular Cancer Therapeutics* **2011,** 10, (11), 2189-2199.
38. Chen, D.; Mao, C.; Zhou, Y.; Su, Y.; Liu, S.; Qi, W. Q., PF-04691502, a dual PI3K/mTOR inhibitor has potent pre-clinical activity by inducing apoptosis and G1 cell cycle arrest in aggressive B-cell non-Hodgkin lymphomas. *International journal of oncology* **2016,** 48, (1), 253-60.
39. Bresin, A.; Cristofoletti, C.; Caprini, E.; Cantonetti, M.; Monopoli, A.; Russo, G.; Narducci, M. G., Preclinical Evidence for Targeting PI3K/mTOR Signaling with Dual-Inhibitors as a Therapeutic Strategy against Cutaneous T-Cell Lymphoma. *J Invest Dermatol* **2019**.
40. Knight, S. D.; Adams, N. D.; Burgess, J. L.; Chaudhari, A. M.; Darcy, M. G.; Donatelli, C. A.; Luengo, J. I.; Newlander, K. A.; Parrish, C. A.; Ridgers, L. H.; Sarpong, M. A.; Schmidt, S. J.; Van Aller, G. S.; Carson, J. D.; Diamond, M. A.; Elkins, P. A.; Gardiner, C. M.; Garver, E.; Gilbert, S. A.; Gontarek, R. R.; Jackson, J. R.; Kershner, K. L.; Luo, L.; Raha, K.; Sherk, C. S.; Sung, C.-M.; Sutton, D.; Tummino, P. J.; Wegrzyn, R. J.; Auger, K. R.; Dhanak, D., Discovery of GSK2126458, a Highly Potent Inhibitor of PI3K and the Mammalian Target of Rapamycin. *ACS Medicinal Chemistry Letters* **2010,** 1, (1), 39-43.
41. Ippolito, T.; Tang, G.; Mavis, C.; Gu, J. J.; Hernandez-Ilizaliturri, F. J.; Barth, M. J., Omipalisib (GSK458), a Novel Pan-PI3K/mTOR Inhibitor, Exhibits In Vitro Anti-Lymphoma Activity in Chemotherapy-Sensitive and -Resistant Models of Burkitt Lymphoma. *Blood* **2016,** 128, (22), 5376-5376.
42. Gu, J.; Yang, L.; Gaughan, D. C.; He, L.; Shen, W.; Mavis, C.; Hernandez-Ilizaliturri, F. J., GSK458 Is a Novel Dual PI3K/mTOR Inhibitor with Preclinical Antitumor Activity in T Cell Lymphomas As a Single Agent and in Combination Therapy. *Blood* **2018,** 132, (Supplement 1), 5378-5378.
43. Hart, S.; Chng, W.-J., A Novel and Highly Selective Dual PI3K/mTOR Kinase Inhibitor VS-5584, Shows Promising Therapeutic Potential For The Treatment Of Multiple Myeloma. *Blood* **2013,** 122, (21), 4433-4433.
44. Poulsen, A.; Nagaraj, H.; Lee, A.; Blanchard, S.; Soh, C. K.; Chen, D.; Wang, H.; Hart, S.; Goh, K. C.; Dymock, B.; Williams, M., Structure and ligand-based design of mTOR and PI3-kinase inhibitors leading to the clinical candidates VS-5584 (SB2343) and SB2602. *J Chem Inf Model* **2014,** 54, (11), 3238-50.
45. Leahy, J. J.; Golding, B. T.; Griffin, R. J.; Hardcastle, I. R.; Richardson, C.; Rigoreau, L.; Smith, G. C., Identification of a highly potent and selective DNA-dependent protein kinase (DNA-PK) inhibitor (NU7441) by screening of chromenone libraries. *Bioorg Med Chem Lett* **2004,** 14, (24), 6083-7.
46. Tomska, K.; Kurilov, R.; Lee, K. S.; Hullein, J.; Lukas, M.; Sellner, L.; Walther, T.; Wagner, L.; Oles, M.; Brors, B.; Huber, W.; Zenz, T., Drug-based perturbation screen uncovers synergistic drug combinations in Burkitt lymphoma. *Sci Rep* **2018,** 8, (1), 12046.
47. Koul, D.; Wang, S.; Wu, S.; Saito, N.; Zheng, S.; Gao, F.; Kaul, I.; Setoguchi, M.; Nakayama, K.; Koyama, K.; Shiose, Y.; Sulman, E. P.; Hirota, Y.; Yung, W. K. A., Preclinical therapeutic efficacy of a novel blood-brain barrier-penetrant dual PI3K/mTOR inhibitor with preferential response in PI3K/PTEN mutant glioma. *Oncotarget* **2017,** 8, (13), 21741-21753.
48. Heffron, T. P.; Berry, M.; Castanedo, G.; Chang, C.; Chuckowree, I.; Dotson, J.; Folkes, A.; Gunzner, J.; Lesnick, J. D.; Lewis, C.; Mathieu, S.; Nonomiya, J.; Olivero, A.; Pang, J.; Peterson, D.; Salphati, L.; Sampath, D.; Sideris, S.; Sutherlin, D. P.; Tsui, V.; Wan, N. C.; Wang, S.; Wong, S.; Zhu, B. Y., Identification of GNE-477, a potent and efficacious dual PI3K/mTOR inhibitor. *Bioorg Med Chem Lett* **2010,** 20, (8), 2408-11.
49. Cheng, H.; Li, C.; Bailey, S.; Baxi, S. M.; Goulet, L.; Guo, L.; Hoffman, J.; Jiang, Y.; Johnson, T. O.; Johnson, T. W.; Knighton, D. R.; Li, J.; Liu, K. K.; Liu, Z.; Marx, M. A.; Walls, M.; Wells, P. A.; Yin, M. J.; Zhu, J.; Zientek, M., Discovery of the Highly Potent PI3K/mTOR Dual Inhibitor PF-04979064 through Structure-Based Drug Design. *ACS Med Chem Lett* **2013,** 4, (1), 91-7.
50. Venkatesan, A. M.; Chen, Z.; dos Santos, O.; Dehnhardt, C.; Santos, E. D.; Ayral-Kaloustian, S.; Mallon, R.; Hollander, I.; Feldberg, L.; Lucas, J.; Yu, K.; Chaudhary, I.; Mansour, T. S., PKI-179: an orally efficacious dual phosphatidylinositol-3-kinase (PI3K)/mammalian target of rapamycin (mTOR) inhibitor. *Bioorg Med Chem Lett* **2010,** 20, (19), 5869-73.

51. Mallon, R.; Hollander, I.; Feldberg, L.; Lucas, J.; Soloveva, V.; Venkatesan, A.; Dehnhardt, C.; Delos Santos, E.; Chen, Z.; Dos Santos, O.; Ayral-Kaloustian, S.; Gibbons, J., Antitumor efficacy profile of PKI-402, a dual phosphatidylinositol 3-kinase/mammalian target of rapamycin inhibitor. *Mol Cancer Ther* **2010,** 9, (4), 976-84.
52. Dehnhardt, C. M.; Venkatesan, A. M.; Delos Santos, E.; Chen, Z.; Santos, O.; Ayral-Kaloustian, S.; Brooijmans, N.; Mallon, R.; Hollander, I.; Feldberg, L.; Lucas, J.; Chaudhary, I.; Yu, K.; Gibbons, J.; Abraham, R.; Mansour, T. S., Lead optimization of N-3-substituted 7-morpholinotriazolopyrimidines as dual phosphoinositide 3-kinase/mammalian target of rapamycin inhibitors: discovery of PKI-402. *J Med Chem* **2010,** 53, (2), 798-810.
53. Rageot, D.; Bohnacker, T.; Keles, E.; McPhail, J. A.; Hoffmann, R. M.; Melone, A.; Borsari, C.; Sriramaratnam, R.; Sele, A. M.; Beaufils, F.; Hebeisen, P.; Fabbro, D.; Hillmann, P.; Burke, J. E.; Wymann, M. P., (S)-4-(Difluoromethyl)-5-(4-(3-methylmorpholino)-6-morpholino-1,3,5-triazin-2-yl) pyridin-2-amine (PQR530), a Potent, Orally Bioavailable, and Brain-Penetrable Dual Inhibitor of Class I PI3K and mTOR Kinase. *J Med Chem* **2019,** 62, (13), 6241-6261.
54. Rewcastle, G. W.; Flanagan, J. U.; Giddens, A. C.; Gamage, S. A.; Tsang, S. K.; Kendall, J. D.; Baguley, B. C.; Buchanan, C. M.; Matthews, D. J.; O'Farrell, M.; Jamieson, S. M.; Denny, W. A.; Shepherd, P. R., Abstract 1644: Design and discovery of PWT33597 (VDC-597), a dual inhibitor of PI3-kinase alpha and mTOR. *Cancer research* **2014,** 74, (19 Supplement), 1644-1644.
55. Smith, M. C.; Mader, M. M.; Cook, J. A.; Iversen, P.; Ajamie, R.; Perkins, E.; Bloem, L.; Yip, Y. Y.; Barda, D. A.; Waid, P. P.; Zeckner, D. J.; Young, D. A.; Sanchez-Felix, M.; Donoho, G. P.; Wacheck, V., Characterization of LY3023414, a Novel PI3K/mTOR Dual Inhibitor Eliciting Transient Target Modulation to Impede Tumor Growth. *Mol Cancer Ther* **2016,** 15, (10), 2344-2356.
56. Bendell, J. C.; Varghese, A. M.; Hyman, D. M.; Bauer, T. M.; Pant, S.; Callies, S.; Lin, J.; Martinez, R.; Wickremsinhe, E.; Fink, A.; Wacheck, V.; Moore, K. N., A First-in-Human Phase 1 Study of LY3023414, an Oral PI3K/mTOR Dual Inhibitor, in Patients with Advanced Cancer. *Clinical cancer research : an official journal of the American Association for Cancer Research* **2018,** 24, (14), 3253-3262.
57. Rewcastle, G. W.; Kolekar, S.; Buchanan, C. M.; Gamage, S. A.; Giddens, A. C.; Tsang, K. Y.; Kendall, J. D.; Singh, R.; Lee, W. J.; Smith, G. C.; Han, W.; Matthews, D. J.; Denny, W. A.; Shepherd, P. R.; Jamieson, S. M. F., Biological characterization of SN32976, a selective inhibitor of PI3K and mTOR with preferential activity to PI3Kalpha, in comparison to established pan PI3K inhibitors. *Oncotarget* **2017,** 8, (29), 47725-47740.
58. Giddens, A. C.; Gamage, S. A.; Kendall, J. D.; Lee, W.-J.; Baguley, B. C.; Buchanan, C. M.; Jamieson, S. M. F.; Dickson, J. M. J.; Shepherd, P. R.; Denny, W. A.; Rewcastle, G. W., Synthesis and biological evaluation of solubilized sulfonamide analogues of the phosphatidylinositol 3-kinase inhibitor ZSTK474. *Bioorganic & Medicinal Chemistry* **2019,** 27, (8), 1529-1545.
59. Li, T.; Wang, J.; Wang, X.; Yang, N.; Chen, S. M.; Tong, L. J.; Yang, C. H.; Meng, L. H.; Ding, J., WJD008, a dual phosphatidylinositol 3-kinase (PI3K)/mammalian target of rapamycin inhibitor, prevents PI3K signaling and inhibits the proliferation of transformed cells with oncogenic PI3K mutant. *J Pharmacol Exp Ther* **2010,** 334, (3), 830-8.
60. Jalota-Badhwar, A.; Bhatia, D. R.; Boreddy, S.; Joshi, A.; Venkatraman, M.; Desai, N.; Chaudhari, S.; Bose, J.; Kolla, L. S.; Deore, V.; Yewalkar, N.; Kumar, S.; Sharma, R.; Damre, A.; More, A.; Sharma, S.; Agarwal, V. R., P7170: A Novel Molecule with Unique Profile of mTORC1/C2 and Activin Receptor-like Kinase 1 Inhibition Leading to Antitumor and Antiangiogenic Activity. *Molecular Cancer Therapeutics* **2015,** 14, (5), 1095-1106.
61. Bean, J. R.; Hosford, S. R.; Symonds, L. K.; Owens, P.; Dillon, L. M.; Yang, W.; Shee, K.; Schwartz, G. N.; Marotti, J. D.; Muller, K. E.; Rosenkranz, K. M.; Barth, R. J.; Chen, V. S.; Agarwal, V. R.; Miller, T. W., The PI3K/mTOR dual inhibitor P7170 demonstrates potent activity against endocrine-sensitive and endocrine-resistant ER+ breast cancer. *Breast cancer research and treatment* **2015,** 149, (1), 69-79.
62. Venkatesha, V. A.; Joshi, A.; Venkataraman, M.; Sonawane, V.; Bhatia, D.; Tannu, P.; Bose, J.; Choudhari, S.; Srivastava, A.; Pandey, P. K.; Lad, V. J.; Sangana, R.; Ahmed, T.; Damre, A.; Deore, V.; Sahu, B.; Kumar, S.; Sharma, S.; Agarwal, V. R., P7170, a novel inhibitor of mTORC1/mTORC2 and Activin receptor-like Kinase 1 (ALK1) inhibits the growth of non small cell lung cancer. *Molecular Cancer* **2014,** 13, (1), 259.
63. Gaulton, A.; Hersey, A.; Nowotka, M.; Bento, A. P.; Chambers, J.; Mendez, D.; Mutowo, P.; Atkinson, F.; Bellis, L. J.; Cibrian-Uhalte, E.; Davies, M.; Dedman, N.; Karlsson, A.; Magarinos, M. P.; Overington, J. P.; Papadatos, G.; Smit, I.; Leach, A. R., The ChEMBL database in 2017. *Nucleic Acids Res* **2017,** 45, (D1), D945-D954.
64. Sterling, T.; Irwin, J. J., ZINC 15--Ligand Discovery for Everyone. *J Chem Inf Model* **2015,** 55, (11), 2324-37.
65. Kim, S.; Chen, J.; Cheng, T.; Gindulyte, A.; He, J.; He, S.; Li, Q.; Shoemaker, B. A.; Thiessen, P. A.; Yu, B.; Zaslavsky, L.; Zhang, J.; Bolton, E. E., PubChem 2019 update: improved access to chemical data. *Nucleic Acids Res* **2019,** 47, (D1), D1102-d1109.
66. Wishart, D. S.; Feunang, Y. D.; Guo, A. C.; Lo, E. J.; Marcu, A.; Grant, J. R.; Sajed, T.; Johnson, D.; Li, C.; Sayeeda, Z.; Assempour, N.; Iynkkaran, I.; Liu, Y.; Maciejewski, A.; Gale, N.; Wilson, A.; Chin, L.; Cummings, R.; Le, D.; Pon, A.; Knox, C.; Wilson, M., DrugBank 5.0: a major update to the DrugBank database for 2018. *Nucleic Acids Res* **2018,** 46, (D1), D1074-d1082.

Supplementary Table 1. Chemical structures of dual PI3K/mTOR inhibitors that are not currently under clinical development. Data collected from https://www.ebi.ac.uk/chembl/ [63], http://zinc.docking.org/substances/home/ [64], https://pubchem.ncbi.nlm.nih.gov/ [65], https://www.drugbank.ca/ [66], https://fdasis.nlm.nih.gov/srs/. MW, molecular weight. IUPAC, International Union of Pure and Applied Chemistry. The three dual PI3K/mTOR inhibitors that are still in clinical development for humans (bimiralisib, GDC-0084 and gedatolisib) are presented in Table 1.

Official /Common/ Alternative name	3D-Structure	IUPAC Name	MW
Apitolisib, GDC-0980, RG7422	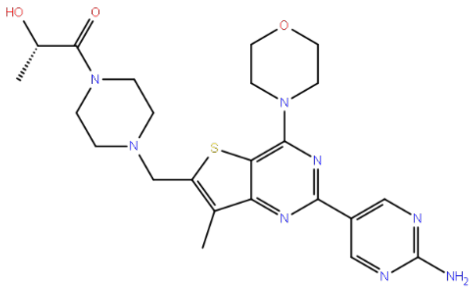	(2S)-1-(4-{[2-(2-aminopyrimidin-5-yl)-7-methyl-4-(morpholin-4-yl)thieno[3,2-d]pyrimidin-6-yl]methyl}piperazin-1-yl)-2-hydroxypropan-1-one	498.61
Dactolisib, BEZ235, NVP-BEZ235, RTB-101, NVP-BEZ235-NX	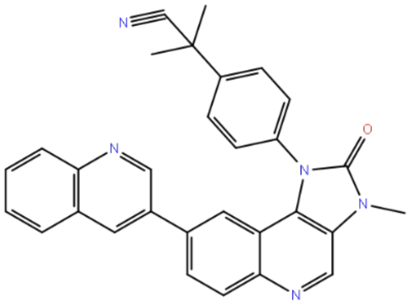	2-methyl-2-{4-[3-methyl-2-oxo-8-(quinolin-3-yl)-1H,2H,3H-imidazo[4,5-c]quinolin-1-yl]phenyl}propanenitrile	469.55
Omipalisib, GSK2126458, GSK458, GSK-212	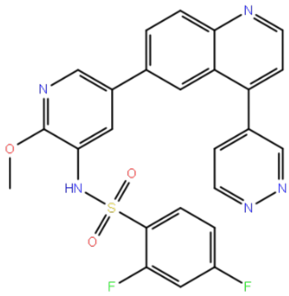	2,4-difluoro-N-{2-methoxy-5-[4-(pyridazin-4-yl)quinolin-6-yl]pyridin-3-yl}benzene-1-sulfonamide	505.51
Panulisib, P7170, S9WA04F921	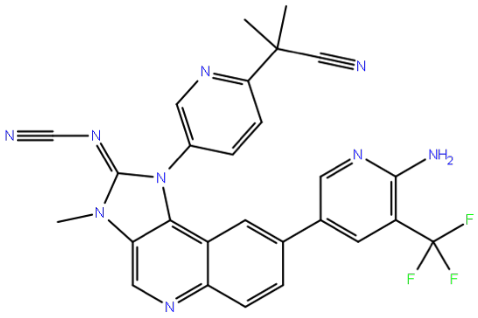	[8-[6-amino-5-(trifluoromethyl)pyridin-3-yl]-1-[6-(2-cyanopropan-2-yl)pyridin-3-yl]-3-methylimidazo[4,5-c]quinolin-2-ylidene]cyanamide	527.5

Official /Common/ Alternative name	3D-Structure	IUPAC Name	MW
Samotolisib, LY3023414, GTPL8918	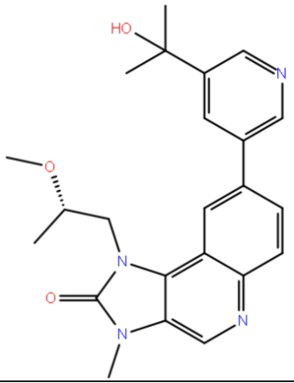	8-[5-(2-hydroxypropan-2-yl)pyridin-3-yl]-1-[(2S)-2-methoxypropyl]-3-methylimidazo[4,5-c]quinolin-2-one	406.5
Voxtalisib, XL765, SAR245409	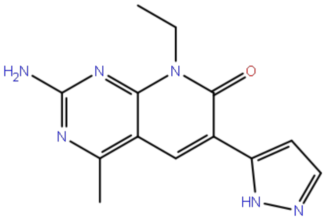	2-amino-8-ethyl-4-methyl-6-(1H-pyrazol-5-yl)-7H,8H-pyrido[2,3-d]pyrimidin-7-one	270.3
BGT226, NVP-BGT226	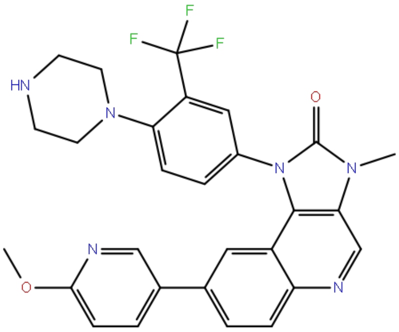	8-(6-methoxypyridin-3-yl)-3-methyl-1-[4-piperazin-1-yl-3-(trifluoromethyl)phenyl]imidazo [4,5-c] quinolin-2-one	534.54
DS7423, 70895382	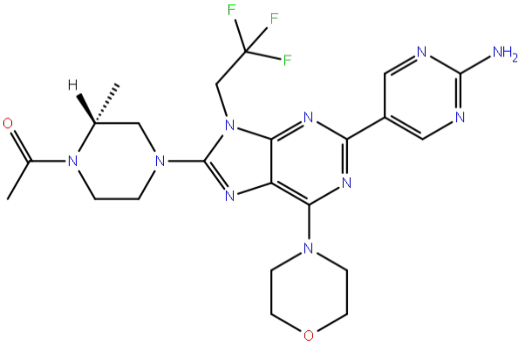	1-{4-[2-(2-aminopyrimidin-5-yl)-6-(morpholin-4-yl)-9-(2,2,2-trifluoroethyl)-9H-purin-8-yl]-2-methylpiperazin-1-yl}ethan-1-one	520.5

Official /Common/ Alternative name	3D-Structure	IUPAC Name	MW
GNE-477	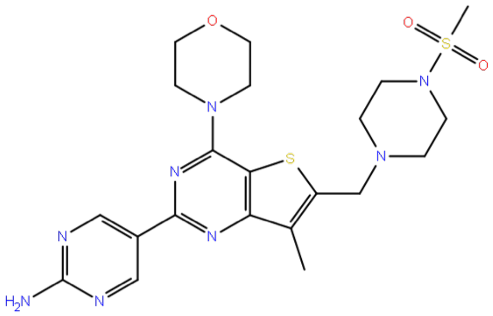	5-(7-methyl-6-((4-(methylsulfonyl)piperazin-1-yl)methyl)-4-morpholinothieno[3,2-d]pyrimidin-2-yl)pyrimidin-2-amine	504.64
PF-04691502	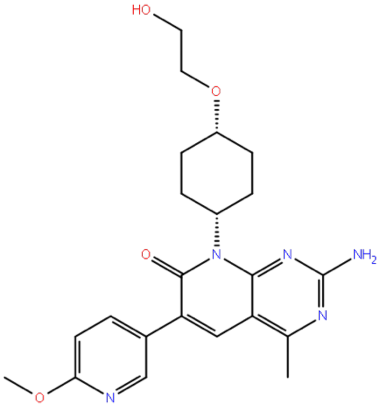	2-amino-6-(6-methoxypyridin-3-yl)-4-methyl-8-[(1r,4r)-4-(2-hydroxyethoxy)cyclohexyl]-7H,8H-pyrido[2,3-d]pyrimidin-7-one	425.49
PF-04979064	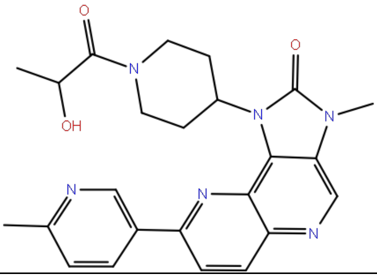	1-[1-[(2S)-2-hydroxypropanoyl]piperidin-4-yl]-3-methyl-8-(6-methylpyridin-3-yl)imidazo[4,5-c][1,5]naphthyridin-2-one	446.51
PI-103, 9884685	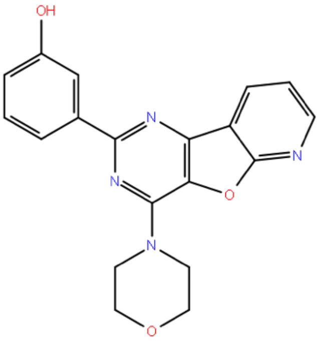	3-(6-morpholin-4-yl-8-oxa-3,5,10-triazatricyclo[7.4.0.02,7]trideca-1(9),2(7),3,5,10,12-hexaen-4-yl)phenol	348.36

Official /Common/ Alternative name	3D-Structure	IUPAC Name	MW
PKI-179	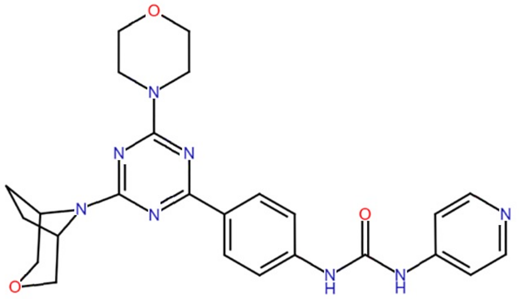	3-{4-[4-(morpholin-4-yl)-6-[(1R,5S)-3-oxa-8-azabicyclo[3.2.1]octan-8-yl]-1,3,5-triazin-2-yl]phenyl}-1-(pyridin-4-yl)urea	488.55
PKI-402, 44187953	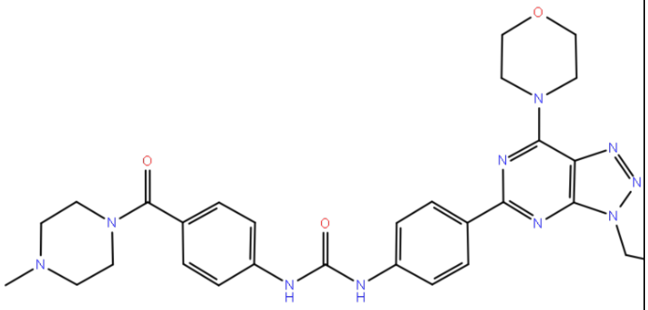	1-[4-(3-ethyl-7-morpholin-4-yltriazolo[4,5-d]pyrimidin-5-yl)phenyl]-3-[4-(4-methylpiperazine-1-carbonyl)phenyl]urea	570.66
PQR530	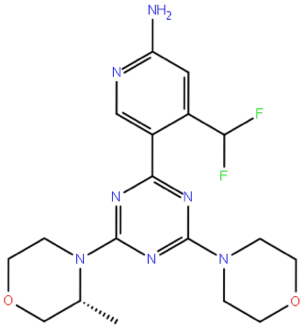	4-(difluoromethyl)-5-[4-[(3S)-3-methylmorpholin-4-yl]-6-morpholin-4-yl-1,3,5-triazin-2-yl]pyridin-2-amine	407.4
PWT33597, VDC-597	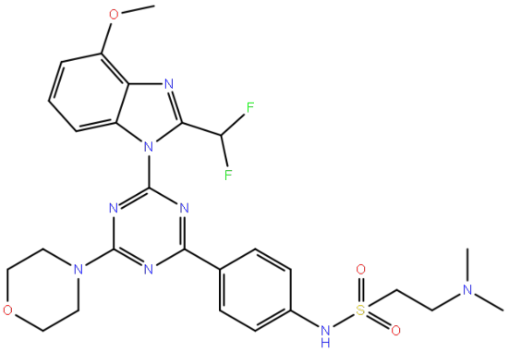	3-{4-[3-ethyl-7-(morpholin-4-yl)-3H-[1,2,3]triazolo[4,5-d]pyrimidin-5-yl]phenyl}-1-[4-(4-methylpiperazine-1-carbonyl)phenyl]urea	684.74
SF-1126		(2S)-2-[[(2S)-3-carboxy-2-[[2-[[(2S)-5-(diaminomethylideneamino)-2-[[4-oxo-4-[[4-(4-oxo-8-phenylchromen-2-yl)morpholin-4-ium-4-yl]methoxy]butanoyl]amino]pentanoyl]amino]acetyl]amino]propanoyl]amino]-3-hydroxypropanoate	852.86

Official /Common/ Alternative name	3D-Structure	IUPAC Name	MW
	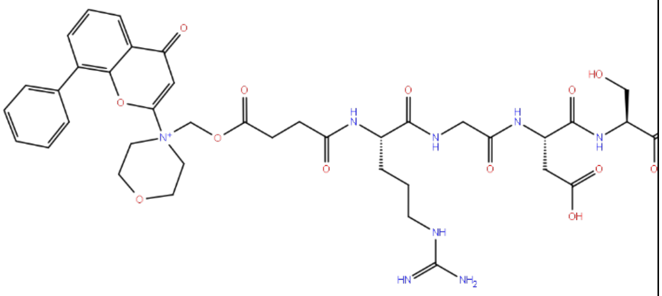		
SN32976, 1246202-11-8	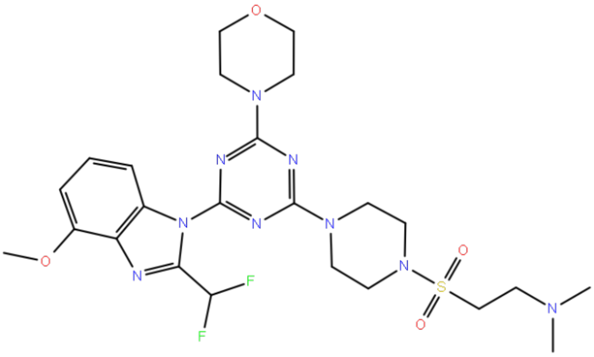	2-[4-[4-[2-(difluoromethyl)-4-methoxybenzimidazol-1-yl]-6-morpholin-4-yl-1,3,5-triazin-2-yl]piperazin-1-yl]sulfonyl-N,N-dimethylethanamine	581.6
VS-5584, SB2343	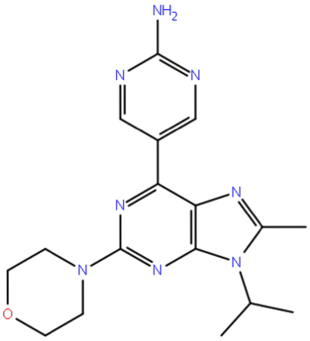	5-(8-methyl-2-morpholin-4-yl-9-propan-2-ylpurin-6-yl)pyrimidin-2-amine	354.4
